# Investigating the status of transgenic crops in Iran in terms of cultivation, consumption, laws and rights in comparison with the world

**DOI:** 10.1038/s41598-021-88713-7

**Published:** 2021-04-28

**Authors:** Abolfazl Baghbani-Arani, Mona Poureisa, Hossein Alekajbaf, Rezvan Karami Borz-Abad, Khodadad Khodadadi-Dashtaki

**Affiliations:** 1 Department of Agriculture Science, Payame Noor University, Tehran, Iran; 2 Department of Law, Payame Noor University, Tehran, Iran

**Keywords:** Biotechnology, Plant biotechnology, Agricultural genetics

## Abstract

Recently, there has been a development in transgenic technologies in many countries to meet nutritional needs of increasing worlds҆ population. However, there are some concerns about possible risks in the field of growing genetically modified (GM) food, such as threats of biodiversity and food allergies making their use a challenge. Therefore, the present study was conducted to investigate the economic effects and political scopes of GM foods in production sector and policies made by different countries in the world and Iran. Moreover, essential (practical and legal) solutions and guidelines were provided for production and consumption of GM foods, which are useful for governmental entities, Iranian politicians, and consumers' rights. The latest situation of transgenic crops in the countries with which Iran has the highest exchange of agricultural products (including Turkey, Pakistan, and the European Union (EU)) was also studied. Although, Iran has been one of leading Asian countries not only in the field of transfer of technical knowledge of genetic engineering, but also in development of the specialized knowledge of biosafety, and despite production of several transgenic plant lines by Iranian researchers, unfortunately no GM crop has obtained release and cultivation license except for GM rice that its growing process was banned after change of government. According to findings of this study, in Iran, growing and production process of GM crops does not follow the global trend owing to scientific and legal infrastructures.

## Introduction

Food security is one of the most significant human challenges in facing with population growth and climate change risks. Now, worlds҆ population is about 7.5 billion people increasing about 83 million people annually; however, it has been estimated to reach about 8.5 and 9.7 billion people by 2030 and 2050, respectively. Such rise in worlds҆ population is the key reason for global poverty^1^. Therefore, eradication of starvation should be a policy priority for countries. Now, increase in crops҆ yield in the cultivated area may be the most realistic solution to meet increasing global demand for crops. However, some countries, such as Iran have limited capacity to expand the area under cultivation^2^. Therefore, more agricultural inputs, such as fertilizers, water, pesticides, or genetic improvers should be used to achieve higher yield per hectare^3^. In this regard, there are several other complex factors including increasing demand for biofuels and production of raw materials and global warming, accelerated urbanization, desertification, salinization and erosion of arable soil, land use change in accordance with economic considerations, climate change, and limited water resources that need to be considered^1^. Scientific innovations in plant biotechnology (genetic engineering) and quantitative advances in farm management are powerful tools used to cope with the above-mentioned challenges.

Genetically modified organisms (GMO) have been defined as the organisms (i.e., plants, animals, or microorganisms), in which the genetic material (DNA) has been altered in a way that does not occur naturally by natural recombination or mating by the world health organization (WHO), food and agriculture organization (FAO), and European Commission. This definition tries to present a new concept of direct manipulation of genetic organisms, differing from conventional performance of improving genetic traits of plants and animals implemented through selective modification over thousands of years.

Some environmental stresses, such as drought, hot or cold weather, salinity of water or soil may lead to economic damage to plants as well as significant decline (up to 70%) in yield of crops, fruits, and vegetables^4^. It is possible to prevent a severe reduction in yield in stressful environments by identifying the genes associated with resistance to these stresses and transferring them to crops^5^. On the other hand, most of GM species are resistant against herbicide or pesticide. Accordingly, it would be possible to remove pesticides or reduce their use in farming; definitely leading to economic and environmental advantages^2,6^. Besides, while reducing the amount of pesticide, production of herbicide-resistant transgenic plants enables the farmer to use no-till system more easily^2,5,7^. As a result, it reduces the amount of eroded soil, provides more protection for plants, reduces the use of farm equipment and fuel consumption and finally, causes less emission of greenhouse gases in the area under cultivation of GM crops^8^. Modifying chemical composition of food to eliminate malnutrition^1,9^, improving food processing^1,10–13^ and finally, using genetic engineering to produce plants that can be used as oral vaccines^14–16^ are another benefits of transgenic plants. On the other hand, because this technology works with living organisms, the risks are more dangerous and unpredictable in interaction with other living organisms than experiments, in which chemicals are used^12,17^.

Religious reasons may be also expressed as concerns about GM foods. According to Raman^18^, there are various orders given by different religions about these foods. Halakha (Jewish Law) has accepted genetic engineering as a method to improve quality or increase amount of food in the world; while there is no consensus on advantages of GM foods or any study on consuming these foods in Christianity. From Islamic viewpoint, there is no need to modify foods genetically as the God has created anything in its perfect form so, human is not allowed to manipulate anything created by divine wisdom^18^. However, according to a previous study, great imitation authorities and Leader of Islamic Republic of Iran have stated that GM foods can be consumed if are not harmful for health^19^.

GM plants have been marketed so far as foodstuffs about one decade after producing the first generation of GM plants in laboratory^20^. In many countries, where farmers are free to choose technology, conventional crops have been replaced with GM plants. For example, corn, cotton, and soybean can be named as GM crops in USA with more than 90% of acceptance for biotechnological products. The same case has occurred for soybean in Brazil and Argentina, cotton in India and China, and canola in Canada^20^. There has been a considerable rise in acceptance of pesticide-resistant GM sugar beets in USA. Farmers could reduce frequent spraying and subsequently, save more time and money by controlling weeds growing in areas under cultivation of pesticide-resistant sugar beets. As this method is cost-effective, farmers have accepted different kinds of biotechnology within a short time^21^.

Many studies have been done to figure out why farmers prefer GM crops to conventional ones. Klümper and Qaim reported on average 68 and 22% increase in farmers' profits and product yield, respectively and 39% reduction in cost of using pesticides after using biotechnology-produced crops^22^. In general, such increased yield and profit was higher in developing countries compared to developed ones. Therefore, farmers achieve an economic profit despite high cost of seeds used for biotechnological plants. In addition to economic advantages, farmers justify the use of such plants due to other non-financial benefits, such as saving time, ease of use, and more flexible planning^23,24^.

About 70–90% of the produced GM crops in the world are used to feed livestock^25^. In the USA, with high acceptance rate of these products, more than 95% of animals, used as human foods are feed by GM plants and more than 100 billion livestock have been fed over the past decade^26^. Health and performance of the livestock have been also studied and no adverse effect caused by feeding livestock with GM crops has been found compared to ordinary foods given to animals^26^. Non-governmental organizations (NGOs) and environmentalists have identified disadvantages for these products including threat to biodiversity, food allergies, etc. based on some unproven studies causing challenges in acceptance of these crops. For increasing level of trust and awareness of the people about consumption of these products, several international institutions, such as the US food and drug administration (FDA) and WHO have monitored these products and their possible side effects, and they must be approved by these regulatory agencies before consumption and distribution.

There is a low global nutritional demand for using the unapproved GM plants. Market share of the unapproved GM soybean has been estimated to be lower than 4.4% and about 7% of the transacted corns are unapproved. Extensive acceptance rate of transgenic varieties in major exporting countries indicates that more than 90% of the produced soybeans in the world are genetically modified. In the European Union (EU), majority of soy-based livestock feed also contains GM components^26,27,50^.

## Methodology for data collection and definitions

This review survey was done by searching the related papers published during 1983—2021 in databases of Google Scholar, SID, IranMedex, Medline, PubMed, Springer, Science Direct, ProQuest, Magiran, ministry of science, research, and technology (MSRT) journals system, Irans҆ medical journals information system, and Islamic world science citation center (ISC) using keywords including food, security, societys҆ health, situation of GM crop in Iran, producers҆ and consumers҆ rights, and labeling.

## Genetic modification and the associated standards in the world and Iran

Many international organizations have paid attention to challenging issue related to consumption of GM products by proposing some principles, standards, and guidelines to increase safety and decrease concerns about these products. Cartagena Protocol on Biosafety can be named as the most important international binding standard and now, 158 countries (such as Iran) are parties to the protocol^27^. Cartagena Protocol on Biosafety associated with convention on biological diversity (CBD) is an international convention ruling over behavior of GMOs resulting from modern biotechnology from one country to another one. This convention was accepted as an agreement attached to CBD. According to the Cartagena Protocol on Biosafety, all the GMOs should be tested in terms of laboratory analysis, greenhouse, and field assessments for product specifications, considered use, environmental effects, and possible risks for humans҆ and animals҆ health to make decision on their application, import, and export. Countries should follow these regulations, approve safe use of GMOs, adopt the required measures through control process and transportation, and enact some rules on labeling and packing of the GMOs. This protocol has predicted some limits on regulations of free trade of GMOs through signing agreements with the world trade organization (WTO)^28^. In the Cartagena Protocol on Biosafety, biosafety measures are done based on the possible interaction between GMOs and environment to preserve biological diversity. For ensuring biosafety, pre-announced agreement is signed between exporting and importing countries. All the countries are informed about converting food waste into animal feed and processed foods. Some statements and documentation should be inserted on the label or next to the label of these products clearly indicating the following sentence "may contain GMOs" and it should be noted that they should not be left in the environment. Applicable regulations on GMOs are declared to countries and they can share this information with other countries. Following Iran's accession to the Cartagena Protocol on Biosafety, the process of drafting the Biosafety Law of the Islamic Republic of Iran began and finally, in 2009, this law was approved by the Islamic Parliament. Not only this law has allowed performing all the affairs associated with GMOs but also it has obliged the government to facilitate release, cultivation, production, consumption, export, and import of GM products regarding local technology based on legal regulations^29,30^. Hence, an organization called as “Biosafety Council” was established in Iran by forming a secretariat in Department of Environment responsible for producing and supplying GM products^31^.

## The latest status of transgenic crops in the world

**Figure 1:**
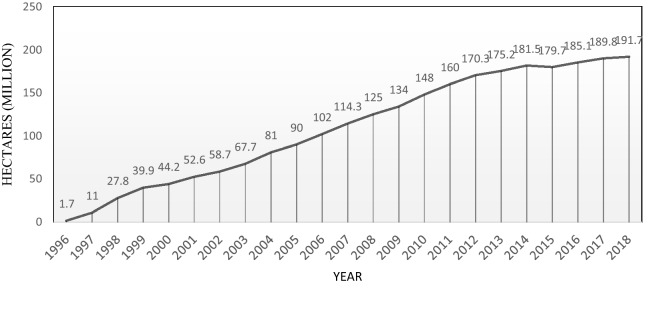
Global Area of Biotech Crops, 1996 to 2018^33^.

There has been a 112-fold increase in the land area under cultivation of transgenic products from the beginning of commercialization in 1996 to the end of 2018 reaching from 1.7 to 189.8 million hectares (Fig. 1). For achieving the increase in yield of transgenic plants, it is estimated that more than 300 million hectares of conventional crops are needed^32^. This additional area should include the lands requiring more water and fertilizers or tropical forests that should be destroyed for this purpose. Such destruction leads to serious environmental concerns globally. Statistics show a global rise in production of GM products so that, 26 countries^7,19^ cultivated GM plants in 2016 and 6.99 and 5.85 million hectares of lands in developing and developed countries were allocated to GM products, respectively. The highest rate of cultivation area was allocated to soybean with 91.4 million hectares accounting for about 50% of total areas used for GM plants.

**Figure 2:**
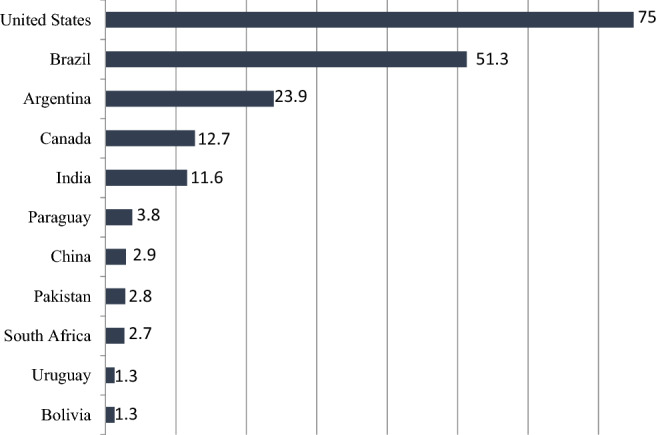
Area of genetically modified (GM) crops worldwide in 2018, by country (in million hectares)^33^.

**Figure 3:**
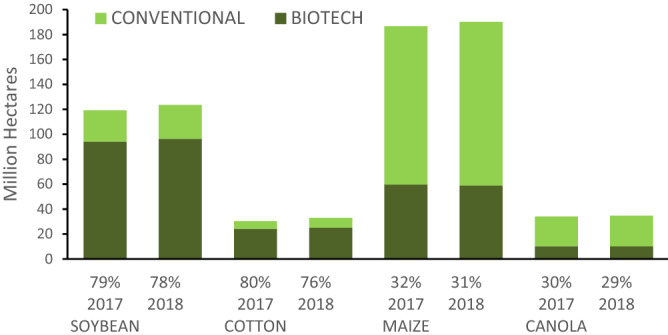
Global area and adoption rate (%) for top 4 Biotech crops (Million hectares) in 2017 and 2018^33^.

Among 26 countries cultivated biotechnology products in 2018, 18 countries have been the pioneers of this trend that have increased the rate by allocating 50.000 hectares of lands to such products. USA is the top producer of biotechnology products in the world by allocating 75 million hectares to these products covering 39% of global crops under cultivation by the biotechnology method; and Brazil is ranked as the second country by allocating 51.3 million hectares (about 27% of global production) (Fig. 2). Soybean, corn, cotton, and canola were the most cultivated biotechnology products in 2018. Although, only a 2% increase has been reported in cultivation of GM soybean (2018 compared to 2017), but an acceptance rate above 50% has been maintained accounting for about 95.9 million hectares of lands. These areas include about 78% of total soybean production in the world (Fig. 3). In 2018, 58.9 million hectares (30% of global production) were allocated to cultivation of GM corn.

Average acceptance rate of GM crops (soybean, corn, and canola) was about 93.3, 93, 100, 92.5, and 95% in USA, Brazil, Argentina, Canada, and India, respectively in 2018^33^. Now, this industry is experiencing a recession period, constant growth of which depends on deregulation in new markets, and research and development of new products. In 2019, 43 GM plants were approved covering 40 species all around the world (Table 1)^34,35^. Number of the modified and approved GM products has been reduced compared to 2 years ago. In 2019, adoption rate of transgenic crops in the world varied from 100% of acceptance in Argentina to its relative acceptance in Asian countries (Pakistan), relative restriction in Europe, and its absolute ban in “Turkey” neighboring Iran Studying this case in the countries exchanging agricultural products with Iran can help to determine the current status of adoption of transgenic crops in Iran.

**Table 1:** New GM product approved in 2019 and the latest update in 2020^34,35^.

Products	GM Trait	Country/region	Approved use
Maize	Glufosinate & Glyphosate & 2,4-D HT, Lepidopteran IR, MM	South Korea	Food use
Maize	Glyphosate & 2,4-D HT	Argentina	Food use, Cultivation
Soybean	Glyphosate & 2,4-D HT	Philippines	F&F
Maize	Glufosinate & Glyphosate & 2,4-D HT, Lepidopteran & Coleopteran IR	EU	F&F
Soybean	Glufosinate & Glyphosate & 2,4-D HT	China, Philippines	F&F
Soybean	Glyphosate HT, Drought stress tolerance	Argentina	Food use, Cultivation
		Brazil	F, F&C
Maize	Glufosinate HT, Coleopteran IR, Multiple IR	Japan	Feed use, Cultivation
		Taiwan	Food use
Maize	Glufosinate & Glyphosate HT, Lepidopteran IR, Multiple IR	EU	F&F
Maize	Glyphosate HT, Lepidopteran IR, MM	Argentina, Brazil	F, F&C
Cotton	Hemipteran IR	Japan, Taiwan	Food use
Canola	Glyphosate HT	China	F&F
Soybean	Lepidopteran IR	EU	F&F
Canola	Modified oil/fatty acid, Imazamox HT	United States	Cultivation
Cotton	Glyphosate & Isoxaflutole HT	Brazil	F, F&C
		Taiwan	Food use
Cotton	Glufosinate & Isoxaflutole HT, Lepidopteran IR	Brazil	F, F&C
Canola	Glufosinate HT, Fertility restoration	China	F&F
Soybean	Glufosinate & Mesotrione HT	China	F&F
Soybean	Glyphosate & Isoxaflutole HT	China	F&F
		Argentina	Food use, Cultivation
Cotton	Glufosinate & Glyphosate HT, Lepidopteran IR, AR, Visual marker	EU	F&F
Maize	Increased Ear Biomass	EU	F&F
Cowpea	Lepidopteran IR	Nigeria	F, F&C
Apple	Non-Browning Phenotype, AR	United States	F, F&C
Sugarcane, (CTC91087-6)	Lepidopteran IR	Brazil	F, F&C
Cotton	AR, Low Gossypol	United States	F, F&C

*F*&*F* food and feed use; *F, F*&*C* food, feed and cultivation; *HT* herbicide tolerant; *IR* insect resistant; *MM* Mannose metabolism; *AR* antibiotic resistance.

## Statistics about Irans҆ trade exchange

In the first 6 months of 2021, major importers of agricultural products and food industries to Iran in terms of import value included Turkey ($692.2), India ($595.4), UAE ($553.3), United Kingdom ($447.2), Russian Federation ($430.7), Netherlands ($268.1), Germany ($185.3), Brazil ($114.8), Singapore ($111.4), Pakistan ($85.1), and Switzerland ($ 69.8 million), which is 86% of the total value of our country's imports. During this period, Iran's largest export destinations for agricultural products and food industry (in terms of export value) were Iraq ($745.8), Afghanistan ($317.6), UAE ($161.8), Russian Federation ($146.9), Pakistan ($119.2), China ($82.8), Turkey ($74), Germany ($57.5), Hong Kong ($56.2), India ($45.5), Kazakhstan ($33.9), Qatar ($32) ,and Oman ($31.1 million ), in total accounting for 89.7% of the total export value of Iran's agricultural and food industries.

## Current position of GM products in Turkey and Pakistan as Iran's neighbours

### Pakistan

Modern biotechnology was performed for the first time in 1985 in Pakistan. So far, 56 advanced biotechnology research institutes (50 public and 6 private organizations) have been established in Pakistan; most of these organizations tend to increase genetic potential. The modified products have been mostly used to help farmers overcoming against biotic and abiotic environmental stresses. GM Cry1Ac-containing cotton (Mon-531) is the only GM product recommended for general cultivation in Pakistan. As Pakistan has signed Cartagena Protocol on Biosafety, national biosafety regulations under the safety rules were approved in 2005 in this country to design research related to development and commercialization of GM products. The license for first GM plant (cotton) was issued in 2010 in Pakistan; and there has been a considerable progress in plants҆ biotechnology and introduction of GM plants in this country. Pests-resistant cotton was introduced in Pakistan then, another version of pests-resistant GM cotton was released in 2012 and two pesticide-resistant and pests-tolerant corn species were introduced in 2017^36^.

### Turkey

Turkey benefits from rich genetic resources so, “biodiversity degradation” is considered as the most important risk for the use of GM products. In Turkey, it is believed that restoring GMOs in nature is possible after their consumption and the lack of information in this field may harm genetic resources. Therefore, this substantial issue should be considered in rules. Production and cultivation of GMOs is forbidden in Turkey. According to Biosafety Act of Turkey, it is banned to produce GM crops. Severe fines have been considered for those who violate this rule in the market. According to Article 15 of Turkish Law entitled as fines, importers or producers of GMOs in the environment are sentenced to 5–12 years of prison and paying a governmental fine of 10.000–200.000 Turkish Liras based on type of the crime committed against the law (not notifying the Biosafety Council). In Turkey, export and import of GMOs is monitored. Monitoring system is based on specific methods and rules for crops with and without genetic alteration. According to this law, GM crops should be labeled based on the decision made by Biosafety Council upon arrival. Therefore, it is not possible to introduce or supply GM crops through ambiguous methods against public opinions; as in Turkey, complete information is included in these products to make consumers aware of the difference between GM and non-GM products. Hence, consumers have the right to choose products. Biosafety Act was introduced in Turkey to solve the problems related to absence of law on GMOs. The combined management of pests and weeds along with integrated control of crops has been considered as an option for genetic usages in Turkey in order to increase quality and efficiency as well as sustainable agricultural development and food safety^10^.

### EU and USA

Considering severe regulatory atmosphere in EU, only one type of GM plant (pests-resistant GM corn) is allowed to be cultivated in the EU. Spain is the only European country with many farms under cultivation of GM products. Farmers have had good experiences and high economic yield regarding efficiency of GM corn compared to ordinary corn in regions contaminated with pests since introduction of this technology in 1998^37^. In 2018, 35 and 6% of total areas of Spain and Portugal (about 121.000 hectares of lands) were allocated to cultivation of GM corn, respectively. While, low amount of GM corn has been cultivated in four other European countries (Portugal, Czech Republic, Romania, and Slovakia).

Although, cultivating pests-resistant GM corn has been approved in EU, but many EU member states have relied on the regulations enacted by EU (412/2015), allowing the countries to ban this technology due to non-scientific reasons^38^. According to reports published by European countries, there is high amount of the imported and consumed GM foods because this ultimately helps in meeting their nutritional demands. On the other hand, when about 80% of the world's soybean crop is transgenic, European countries have to import it, whether they like it or not^24^.

But GM products were widely consumed in livestock feed and food markets in USA market in 1996 after introduction of these products. USA authorities had adopted and approved a permissible policy for GM foods without any need to label GM plants. Furthermore, majority of American consumers were less worried about GM foods and agricultural biotechnology in the years following introduction of these products so that, they used to purchase foodstuffs produced from GM plants despite their limited knowledge about GM foods. There has been a considerable change in social and regulatory framework at both sides of Atlantic Ocean that has directly influenced consumers҆ attitude. In contrary to USA, EU approved strict requirements in this case. Since 1997, in EU, it has been approved to label GM foods- even if genetic modification is identified in final product^39^. Moreover, foods purified from GM plants, such as oils or sugars have been labeled since 2003 even if these products are physically or chemically similar to non-GM products^27,40^. Now, EU has set a certain regulatory framework for cultivating, consuming, and importing GM products for livestock feed and foodstuffs^41,42^. Although, NGOs usually play lesser role in USA, these organizations have performed successfully in introducing GM plants as a threat to biodiversity, farmers' independence, and food safety in Europe^10,24–43^. In general, social and political conditions of Europe justify negative attitude of European consumers toward GM foods compared to consumers in Northern America^27,39–43^. There is a low acceptance on GM plants among European politicians and consumers, in particular in Germany.

## GM crops in Iran (challenges, practical, and legal solutions)

Agriculture sector in Iran has been one of important and relatively stable economic sectors of the country with about 14–15% share of gross domestic product (GDP) despite the unprecedented sanctions over the recent years. Moreover, this sector accounts for about 20% of employment rate in Iran while only 5% of direct employment of individuals in developed countries belongs to agriculture sector. Hence, agriculture sector is the most important part of Iran's economy^44^. Currently, Iran faces with water crisis since about 80–90% of the country's water consumption is related to agricultural purposes^45^. On the other hand, along with the increase in life expectancy, population of Iran is expected to reach 95.3 and more than 112 million people at the lowest and highest predicted levels, respectively in 2031. Therefore, it is essential to develop various crop species with enriched nutrients and high resistance to biotic and abiotic stresses considering increasing population rate, small arable lands, high employment rate in agriculture sector, increasing climate change rate, global warming, and current droughts^28^. Under these conditions, new biotechnology methods including transgenic plants can meet the increase in demand due to population growth without the need for increasing area under cultivation (considering drought and climate change) in Iran.

In Iran, similar oppositions may be seen in NGOs, eco-friendly institutions, and high management levels about GM products. Opponents of development of genetic engineering are trying to prevent from such technology although national law on biosafety forces the government to facilitate release, cultivation, production, consumption, import, and export of GM products. This means that conventional method for production of crops is going to be continued. Iran has been one of leading countries in Asia not only in transferring knowledge of genetic engineering but also in developing biosafety science. Iranian experts participated in the first meetings of advisory committee of the Cartagena Protocol on Biosafety and Iran has been one of the first Asian countries joined this protocol^28^. Some biosafety actions have been done in Iran including establishment of the first scientific biosafety association among Asian countries, designing and approving Biosafety Act, establishing National Biosafety Council, forming the specialized secretariats in three ministries, founding a governmental organization, enforcing assessment mechanism, and issuing licenses regarding environmental release, import, and export of GM products. Although, there have been numerous infrastructures and achievements in the field of commercialization and production of GM products, Iran cannot be named as a successful country in this regard^31^. Despite successful production of several GM plants in field experiments by Iranian researchers, no GM product has received release permission expect for GM rice that was formally released in 2004 but its production was banned after change of government. Lack of scientific governance and national benefits-based approaches to plant genetic engineering as well as debatable issues in this field can be named as reasons for such significant inconsistency between Iran's capacities and achievements on genetic engineering technology^28^. In Iran, more than 99% of crops are produced using conventional agrochemical methods and rate of organic production is lower than 1%. Therefore, chemical toxins are essentially used in Iran's farming technology. Despite dramatic growth in consumption of GM plants in the world, excessive use of chemical pesticides by Iranian farmers, along with legal emphasis on the government's obligation to provide the required facilities for production and consumption of GM plants (Biosafety Law) is still a debatable issue due to opposition of some Iranian managers. Adeli and Ghareyazi^46^ carried out a study and found that 90% of pesticides are used to control crop pests while these pests are controlled by GM plants in the world. Moreover, reduction of these toxins brings numerous advantages for farmers who use these seeds (regarding humans҆ health and environment). Furthermore, repetitive use of insecticides makes insects resistant to the previous forms. Cultivation of GM rice in Iran is a practical example for controlling Asiatic rice borer. There are several GM rice species that can control this pest. There was a global rise in production of GM products in 1996 and Iran also began to produce Asiatic rice borer-resistant GM rice (while, in conventional agricultural method, the highest amount of toxin is used in rice cultivation to combat it). Such achievement in Iran received a considerable attention from the world as this rice called "Taram Moulaee" is the first GM version of rice released in the world and the first GM product produced in Muslim countries and Middle East, which has reached the farms. Hundreds of Iranian farmers have produced this rice but its cultivation has been banned due to biosafety concerns; since then, farmers have used a higher concentration and new compositions of insecticides- due to resistance of pests and inefficiency of pesticides- to control these pests. Accordingly, these GM seeds could reduce the use of pesticides and environmental contaminations and control natural and useful insects living in rice fields, such as ladybirds and fishes. In particular, these seeds could solve the problems of farmers caused by having contact with such toxins^25^. Monitoring type and number of pesticides used in agricultural products is an important and sensitive issue that is not done in Iran as it should be. Rice cultivation in northern provinces of Iran is one of the main sources for livelihood of farmers. Numerous pests existing in rice fields have led to severe crop loss in this area^47^; hence, it is essential to use pesticides and other chemical toxins in the current rice cultivation so that, 60% of total use of pesticides in Iran belongs to northern provinces dominant cropping pattern of which is rice^48^. Reports indicate that pest’s control is usually done using chemical toxins among farmers in north of Iran. Excessive use of pesticides is harmful for useful living insects and organisms in farms so that, they may cause high incidence rate of gastrointestinal cancer in these provinces. On the other hand, chemical pesticides are using increasingly due to their economic benefits, availability, efficiency, and flexibility and there might be no possible reduction in their use rate^46^. In addition, their effectiveness in controlling pests indicates their acceptable function; hence, farmers tend to use chemical pesticides because they are not aware of their negative effects on humans҆ health and environment^48^. Since, the Asiatic rice borer causes 4–6% of damage to the produced rice and 2.9 million tons of rice was produced in 2018 (with 36 kg consumption per capita), 1,000 tons of rice should be imported to keep market equilibrium and support strategic storage of the country^49^. Accordingly, this rate of GM rice varieties (Bt) can supply strategic rice reserves and prevent from rice import and currency outflow. Iran became one of producers of GM products in 2004. Although, production of GM rice was stopped in 2006, 2 GM goats called as Shangool and Mangool were born by diligent researchers in the Royan Institute under supervision of Iran’s Supreme Leader in 2009 and this was a success for researchers of modern biotechnology in agriculture sector^50^. Iranian researchers have achieved some successes in the field of GM plants including Bt gene transfer to Iranian Rice (Taram Moulaee), production of GM cotton and potato, and pests-resistant gene transfer to sugar beet and alfalfa. Tohidfar et al., for the first time created GM alfalfa that generates cry3A gene and resists against alfalfa weevil in 2014^51^. In the case of GM animals, Iranian researchers could have access to technology of coagulation factor protein IX existing in milk of Iranian goat for treating the patients with Hemophilia B and another protein in Iranian goat’s milk to generate medicines for treating heart attack^50^.

On the other hand, Iran is importer of oil, forage, and corn sold as GM products in the global market. It should be noted that only about 10% of soybean derivatives including vegetable oils, soybean flour, lecithin, and soybean protein is non-GM in market of many countries. Moreover, GM grains exist in 20% of the marketed cereals and their derivatives, such as starch and cereal flour and more than 90% of these foods are produced and supplied regardless of labeling based on EU processes and standards, which are the most binding rules in the world. GM foods are going to be produced in the future; for instance, rice, sugar, tea, and sugar beet will be added to the list of GM products. However, livestock feed is the main market of GM products^52,53^. On the other hand, Iran is highly dependent on import of the main global GM products (soy, cotton, corn, and canola) and more than 90% of vegetable oil is imported with the highest area under cultivation in the world. According to statistics, about 2,300 thousand tons of total^2^ vegetable oil consuming in Iran include the imported oil and oilseeds (about 2 million and 150,000 tons of soy, 80,000 tons of canola, and about 55 tons of sesame seed). In 2018, 78% of globally cultivated soybean and 29% of canola were genetically modified so that, even EU countries imported the oil produced from GM plants despite the strict rules adopted for GM plants in Europe. Therefore, vegetable oil-importing countries, such as Iran have to import these foodstuffs. On the other hand, more than 8 million tons of livestock corn was imported to Iran in 2019. This livestock feed was exported from 18 countries of the world including USA as a livestock corn exporter. Accordingly, 2,225 tons of livestock corn was imported to Iran from USA, costing US$ 500, 000. USA is the largest producer of GM products in the world accounting for about 30% of global market share in 2019^54^.

In 2019, Ministry of Health and Medical Education of Iran declared only three transgenic GM products including oilseeds of canola, soybean, and corn as allowed products thus, there is no other GM product in Iran’s market and the named products should have been labeled too. It has been also reported that total soybeans imported to Iran are genetically modified and Iranian people are consuming GM plants over 15 years. On the other hand, more than 6 million tons of corn, livestock and poultry feed imported to Iran are genetically modified; however, there is also GM cotton seed for oil production in Iran’s market. Hence, the Head of Department of Environment and the Main Member of Biosafety Council explained that there is no scientific document on risky effects of GM foods on humans҆ health in Iran and there is a global debate stating that excessive pressure on existing resources is riskier than production and consumption of GM foods^55^. Now, Iran’s FDA has predicted the main provisions on import of GM products to Iran as follows; first, the GM product should also be used in producing country and second, the product should have an international license with a transparent GM and genetic manipulation process. For example, even a GM or non-GM corn by-product imported to Iran should have a valid license in both cases. In the case of GM product, it should present a license proving type of GM as well as consumption permission in producing country. A GM product should have an international license obtained from U.S. FDA or European food safety authority (EFSA)^56^. Furthermore, about 50–60% of cotton is imported to Iran while 80% of the lands under cotton cultivation are allocated to GM varieties in the world^34^. Moreover, international organizations, such as FAO, WHO, EU Commission, French academy of medicine (FAM), American medical association (AMA), and American Toxicology Association have assessed safety of foods produced from GM plants and have approved their safety for humans҆ health^28,57^. According to advantages of GM plants for economy and environmental and humans҆ health , the required licenses for cultivation of these products should be given to farmers based on the Biosafety Law approved by Islamic Parliament, global food standards and protocols, and tasks assigned to beneficiaries by Biosafety Act to track and test food security of GM products. Furthermore, consumer has the right to know which product is genetically modified. GM labeling is a solution used to alleviate concerns about these products. Although, there are disagreements on labeling GM foods and microorganisms, it seems that producers of GM foods and microorganisms as well as biotechnology owners will insist on GM labeling as these labels can represent high quality of GM foods^17^. For instance, high oleic acid-containing GM soybean producers claim that their product contains less saturated fat so it is more suitable for consumption. Therefore, the consumer’s trust will be achieved if there is an access to real and neutral information about GM foods and GMOs given to consumers^58^.

As mentioned earlier, religious attitude of individuals may influence acceptance of GM products. This is an important challenge in using results obtained from genetic engineering studies and production of GM plants or organisms consumed by people. Despite the concerns raised in Islamic principles, there is no dissuasive rule for genetic modification in plants and animals. There have been various opinions about consumption of GM products in Islam and there is no consensus on acceptance of these products. According to the research findings and from Islamic experts’ viewpoints, in Islam (Shia religion), consumption of GM products is unrestricted, provided that they are safe and producing health similar to natural foods. In addition, bioethics has been considered by Islamic authorities. They believe that GM products and their relevant studies and technologies are permissible if safety and ethical aspects are respected. Such provisions are based on proper structures and mechanisms. Seemingly, there are suitable structures, such as Department of Environment, Plant Protection Organization, and other executive organizations in Iran playing a vital role in this field. The above-mentioned organizations cooperate with the National Biosafety Council and Biotechnology Development Headquarter. It is hoped to achieve a proper mechanism in executive acts due to approval of National Biosafety Law.

## Conclusion

Level of public awareness about advantages and disadvantages of transgenic plants is low in Iran and relative and temporary acceptance has been achieved due to the existence of Biosafety Law and licenses issued by the Irans҆ FDA to import and consume them based on specific criteria as well as public trust in responsible agencies and supervisors ,but the lack of informed and strong NGOs in the field of public awareness, along with the possibility of expressing resistance towards their consumption with the increase in public awareness necessitates revision of Biosafety Law and also the developed import and export guidelines. Finally, considering consumption of these products in Iran for more than 15 years and the existence of scientific and legal infrastructure, the main decision-makers in the field of transgenic products in Iran are suggested to continue production of transgenic products in a gentle slope. In this regard, rice produced in Iran using high amounts of pesticides and herbicides, as well as major and basic imported products used to feed livestock, such as corn can be good choices to be produced as transgenic crops.

## References

[1] Zhang Ch, Wohlhueter R, Zhang H (2016). Genetically modified foods: A critical review of their promise and problems. Food Sci. Hum. Wellness.

[2] Mohammadi SZ, Yazdanpenah M (2013). Advantages and benefits of transgenic plants. J. Biosaf..

[3] Oliver MJ (2014). Why we need GMO crops in agriculture. Mo Med..

[4] Baghbani-Arani A, Modarres-Sanavy SAM, MashhadiAkbarBoojar M, Mokhtassi Bidgoli A (2017). Towards improving the agronomic performance, chlorophyll fluorescence parameters and pigments in fenugreek using zeolite and vermicompost under deficit water stress. Ind. Crops & Prod..

[5] Kumar K, Gambhir G, Dass A, Kumar Tripathi A, Singh A, Kumar Jha A, Yadava P, Choudhary M, Rakshit S (2020). Genetically modified crops: Current status and future prospects. Planta.

[6] Haji Mohammadi B, Eslami G, Aalaei M, EhramPoush MH, Rezvani ME, Fallahzadeh H, Shirdeli M (2019). Study and comparison of genetically and non-genetically modified rice from view point of possibility of gene transferring in blood of labouraty animal. J. Toloo Behdasht..

[7] Khosrevi S, Tohidfar M (2012). The role of transgenic products in sustainable development. Journal of Biosafety..

[8] Brookes G (2019). Twenty-one years of using insect resistant (GM) maize in Spain and Portugal: Farm-level economic and environmental contributions. GM Crops Food..

[9] Rizzi A, Raddadi N, Sorlini C, Nordgrd L, Nielsen KM, Daffonchio D (2012). The stability and degradation of dietary DNA in the gastrointestinal tract of mammals: implications for horizontal gene transfer and the biosafety of GMOs. Crit. Rev. Food Sci. Nutr..

[10] Kizilaslan N, Yilmaz B (2013). Area of usage and policies of genetically modified organisms in Turkey. Res. Rev. BioSci..

[11] Oakes JV, Shewmaker CK, Stalker DM (1991). Production of cyclodextrins, a novel carbohydrate, in the tubers of transgenic potato plants. Biotechnology.

[12] Gatew H, Mengistu K (2019). Genetically modified foods (GMOs); a review of genetic engineering. J. Life Sci. Biomed..

[13] Tabashnik BE (1994). Evolution of resistance to *Bacillus thuringiensis*. Annu. Rev. Entomol..

[14] Schafer MG, Ross AA, Londo JP, Burdick CA, Lee EH, Travers SEV, de Water PK, Sagers CL (2011). The establishment of genetically engineered canola populations in the US. PLoS ONE.

[15] Ellstrand NPH, Hancock J (1999). Gene flow and introgression from domesticated plants into their wild relatives. Annu. Rev. Ecol. Syst..

[16] Jhansi Rani S, Usha R (2013). Transgenic plants: Types, benefits, public concerns and future. J. Pharm. Res..

[17] Kathryn E, Kemper M, Goddard E (2012). Understanding and predicting complex traits: Knowledge from cattle. Hum. Mol. Genet..

[18] Raman R (2017). The impact of Genetically Modified GM crops in modern agriculture: A review. GM Crops Food.

[19] Allahyari Frad N (2011). A study of Islamic (Shia) views about consumption of genetically modified organisms products. Iran. J. Ethics Med. Hist..

[20] James, C. *Global Status of Commercialized Biotech/GM Crops: 2014. ISAAA Brief No.49*. (ISAAA, Ithaca, 2014). https://www.isaaa.org/resources/publications/briefs/46/.

[21] Dillen K, Demont M, Tillie P, Demont M (2013). Bred for Europe but grown in the US: The case of GM sugar beet. New Biotechnol..

[22] Klümper W, Qaim MA (2014). A Meta-analysis of the impacts of genetically modified crops. PLoS ONE.

[23] Fernandez-Cornejo J, Wechsler S, Livingston M (2014). Mitchell Genetically Engineered Crops in the United States.

[24] Lucht JM (2015). Public acceptance of plant biotechnology and GM crops. Viruses.

[25] Ghareyazie, B. *The Wide Reflection of Production of the First Transgenic Rice by Iran: Reported by Agrifood Awareness: Iran Takes the Biotech Lead. Economic Research Report, 04 6 85 1*. (Center for Strategic Research, 2006)

[26] Van-Eenennaam AL, Young AE (2014). Prevalence and impacts of genetically engineered feedstuffs on livestock populations. J. Anim. Sci..

[27] Mitchell P (2003). Europe angers US with strict GM labeling. Nat. Biotechnol..

[28] Zul Ali, J & Kahak, S. Indigenous technology book of transgenic products (Answers to ten basic questions). Research Center of the Vice President for Science (2019).

[29] Pak, P & Khosravipoor, B. *Transgenic Products: Opportunities and Threats. Conference on Transgenic Products in the Service of Healthy Food Production, Environmental Protection and Sustainable Development* 1–8 (2016).

[30] Dona A, Arvanitoyannis IS (2009). Health risks of genetically modified foods. Crit. Rev. Food Sci. Nutr..

[31] Mohsenpour M, Kahak S, Ghareyazie B (2018). Genetic engineering and food security. Strategic research. J. Agric. Sci. Nat. Resour..

[32] Brookes G, Barfoot P (2016). GM crops: Global Socio-economic and Environmental Impacts 1996–2014.

[33] ISAAA. *Global Status of Commercialized Biotech/GM Crops in 2018: Biotech Crops Continue to Help Meet the Challenges of Increased Population and Climate Change. ISAAA Brief No. 54*. (ISAAA, Ithaca, 2018). https://www.isaaa.org.

[34] Zhang, J. 2019 overview of global GMO development. Agropages 1–11 (2020).

[35] ISAAA. *International Service for the Acquisition of Agri-biotech. GM Approval database*. http://www.isaaa.org/gmapprovaldatabase (2020).

[36] Babar U, Amjad Nawaz M, Arshad U, Tehseen Azhar M, Atif RM, Golokhvast KS, Tsatsakis AM, Shcerbakova K, Chung G, Rana IA (2019). Transgenic crops for the agricultural improvement in Pakistan: A perspective of environmental stresses and the current stat. GM Crops & Food..

[37] Lefebvre L, Polet Y, Williams B (2014). Agricultural Biotechnology Annual Biotechnology and Other New Production Technologies. USDA GAIN Report.

[38] Brookes G (2019). Twenty-one years of using insect resistant (GM) maize in Spain and Portugal: farm-level economic and environmental contributions. GM Crops Food.

[39] Bernauer T, Meins E (2003). Technological revolution meets policy and the market: Explaining cross-national differences in agricultural biotechnology regulation. Eur. J. Polit. Res..

[40] Du L (2014). GMO labelling and the consumer’s right to know: A comparative review of the legal bases for the consumer’s right to genetically modified food labelling. McGill J. Law Health..

[41] Raybould A, Poppy GM (2012). Commercializing genetically modified crops under EU regulations. GM Crops Food.

[42] Varzakas TH, Arvanitoyannis IS, Baltas H (2007). The politics and science behind GMO acceptance. Crit. Rev. Food Sci. Nutr..

[43] Zilberman D, Kaplan S, Kim E, Hochman G, Graff G (2013). Continents divided: Understanding differences between Europe and North America in acceptance of GM crops. GM Crops Food.

[44] Ministry of Agriculture-Jahad. https://www.maj.ir/index.aspx?page_=dorsaetoolseevents&lang (2020).

[45] Saeedi Tehrani S, Parsapour A, Larijani B (2016). Ethical considerations in new genetic technologies with a special focus on transgenic products. Iran. J. Ethics Med. Hist..

[46] Adeli N, Ghareyazie B (2013). Comparison between the impact of transgenic insect resistant crop plants and their traditional counterparts on human health and the environment. Genet. Eng. Biosaf. J..

[47] Abdollahzadeh GhH, Sharif Sharifzadeh M, Qadami Amraei Z (2017). Assessing awareness of rice farmers of Sari County about impacts of usage of pesticides and its health risk in cropping year 2015. Iran. J. Health Environ..

[48] Niyaki A, Radjabi R, Allahyari MS (2010). Social factors critical for adoption of biological control agents Trichogramma spp. egg parasitoid of rice stem borer Chilo suppressalis in North of Iran. Am.-Euras. J. Agric. Environ. Sci..

[49] Statistics of the Ministry of Jihad-Agriculture of Iran. http://anris.agri-peri.ir. (2018).

[50] Ghareyazie B, Mottaqi A, Vishlagh N, Rashedi H (2010). Biosafety at international agreements/organizations. Med. Rights.

[51] Tohidfar M, Zare N, Salhi G, Eftghari M (2013). Agrobacterium-mediated transformation of alfalfa *Medicago sativa* using a synthetic cry3a gene to enhance resistance against alfalfa weevil. Plant Cell Tissue Organ Cult..

[52] Februhartanty J, Widyastuti TN, Iswarawanti DN (2007). Attitudes of agricultural scientists in Indonesia towards genetically modified foods. Asia Pac. J. Clin. Nutr..

[53] Kazemie AH, Abbasi M (2007). Des produits alimentaires génétiquement modifiés et le droit du consommateur. J. Med. Law.

[54] International Service for the Acquisition of Agribiotech Applications. *Are food Derived from GM Crops Safe?*. http://www.isaaa.org (2009).

[55] Anonymous, 2019. https://www.khabaronline.ir/newswards GM crop adoption. Plant Biotechnol. J. **9**, 945–957. 10.1111/j.1467-7652.2011.00651.x 21923717

[56] Anonymous, 2016. https://www.tasnimnews.com/fa/news

[57] James, C. *Global Status of Commercialized Biotech/GM Crops: 2009. ISAAA Brief No. 41*. (ISAAA, Ithaca, 2009). https://www.isaaa.org/resources/publications/briefs/46/.

[58] Delaney B (2007). Strategies to evaluate the safety of bioengineered foods. Int. J. Toxicol..

